# Early Resection of the Tibialis Anterior Tendon for Tendon Exposure After Total Ankle Arthroplasty to Prevent Deep Infection: A Report of Three Cases in Patients With Rheumatoid Arthritis

**DOI:** 10.7759/cureus.40441

**Published:** 2023-06-14

**Authors:** Takaaki Noguchi, Makoto Hirao, Gensuke Okamura, Shigeyoshi Tsuiji, Yuki Etani, Kosuke Ebina, Hideki Tsuboi, Yoshihiko Hoshida, Seiji Okada, Jun Hashimoto

**Affiliations:** 1 Orthopaedic Surgery, National Hospital Organization, Osaka Minami Medical Center, Kawachinagano, JPN; 2 Orthopaedics and Rheumatology, Nippon Life Hospital, Osaka, JPN; 3 Orthopaedic Surgery, Osaka University, Graduate School of Medicine, Suita, JPN; 4 Musculoskeletal Regenerative Medicine, Osaka University, Graduate School of Medicine, Suita, JPN; 5 Orthopaedic Surgery/Rheumatology, Osaka Rosai Hospital, Sakai, JPN; 6 Pathology, National Hospital Organization, Osaka Minami Medical Center, Kawachinagano, JPN

**Keywords:** prevent, deep infection, early resection, tibialis anterior tendon, rheumatoid arthritis, total ankle arthroplasty

## Abstract

Exposure of the tibialis anterior (TA) tendon with wound dehiscence after total ankle arthroplasty (TAA) with the anterior approach is a problematic complication, especially in rheumatoid arthritis (RA) patients. Once the TA tendon is exposed, the duration of wound healing is prolonged, and it could be a risk factor for deep infection. Thus, early resection of the TA tendon was evaluated for tendon exposure with wound dehiscence after TAA in RA patients. In this case report, three rheumatoid ankles that showed wound dehiscence with exposure of the TA tendon after TAA with the anterior approach are presented. Early resection of the TA tendon and debridement under local anesthesia were performed within two days after wound dehiscence. In all cases, wound healing was completed within two weeks after the treatment. Drop foot was not seen in any patients, and there was no difference between the pre and postoperative (1 year after TAA) range of dorsiflexion. Muscle strength for ankle dorsiflexion was also maintained. In conclusion, early resection of the TA tendon appears to be a useful option for undesirable tendon exposure with wound dehiscence to prevent deep infection and prolonged wound healing after total ankle arthroplasty in RA patients.

## Introduction

In recent years, total ankle arthroplasty (TAA) has been chosen for the reconstruction of the rheumatoid ankle with destructive changes. However, superficial infection/delayed wound healing is one of the complications after TAA with the anterior approach, particularly in cases of rheumatoid arthritis (RA) [1]. It has been reported that resection of the tibialis anterior (TA) tendon was useful for refractory tendon exposure with wound dehiscence even though skin treatment was required multiple times [2]. Until resection of the tendon is performed, a superficial infection can progress to a deep infection during skin treatment. Thus, in the present study, early resection of the TA tendon was performed in three cases of tendon exposure with skin dehiscence after TAA in RA cases because it is thought that the risk of deep infection should be eliminated as soon as possible, particularly in RA patients [1,3]. In these three cases, no treatment, including negative pressure wound therapy (NPWT) and/or skin grafting, was performed before tendon resection.

## Case presentation

Case 1

**Figure 1:**
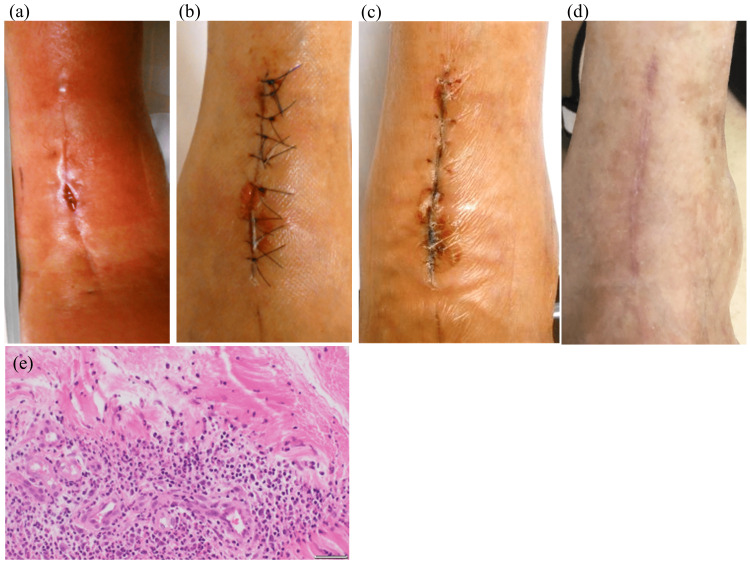
Case 1 (a) Cellulitis occurred 50 days after TAA, and the TA tendon was glimpsed with wound dehiscence. (b) The next day, a resection of the TA tendon was performed. The panel shows the wound six days after resection. (c) Wound healing was complete, and the threads were removed 14 days after the resection. (d) The wound at three months after resection. No skin problems were observed. (e) A histological section of the tibialis anterior tendon (×40) with hematoxylin-eosin (HE) staining was performed. Inflammatory cells, including neutrophils, had infiltrated into the infected tendon tissue. TAA: total ankle arthroplasty, TA: tibialis anterior

A 67-year-old woman with a 25-year history of RA (Steinbrocker classification Class II, stage IV) had a Japan Society for Surgery of the Foot (JSSF) RA foot and ankle scale score of 38 points and a JSSF ankle/hindfoot scale score of 41 points (Table 1). Other procedures for the fore and midfoot deformity were performed previously. She underwent TAA with the anterior approach. Then, 18 days after TAA, wound healing was completed, and the threads were removed. She started to walk with an air-cast (ankle brace) at 21 days and left the hospital at 28 days. Fifty days after TAA, cellulitis was seen around the wound area, and the wound dehisced partially (Figure 1a). The TA tendon was glimpsed, it was not completely exposed, but resection of about 10 cm of the TA tendon with debridement was performed immediately under local anesthesia the next day. Tendon cultures grew Staphylococcus aureus. After resection, wound healing was observed at 14 days, and the threads were removed (Figures 1b, 1c). Three months after resection, she was able to walk without assistance, and the wound was clear (Figure 1d). Her JSSF RA foot and ankle scale score improved from 38 to 69 points, and the JSSF ankle/hindfoot scale score improved from 41 to 67 points one year after TAA (Table 1). At the same time, muscle strength was measured by the manual muscle testing scale (MMT) ranging from 0 to 5 (5: indicating the strongest muscles, able to move against gravity and maximum resistance for the full ROM) [4]. In detail, the patient is short sitting with the ankle in the plantarflexed state. The patient’s leg was stabilized just above the malleoli by the therapist’s hand. The other hand is used for resistance by placing and pushing the dorsal aspect of the foot. The patient tried active dorsiflexion against the resistance. She had five degrees of active dorsiflexion, and muscle strength for ankle dorsiflexion was maintained (manual muscle testing (MMT): on histological examination, inflammatory cells, including neutrophils, had infiltrated into the infected tendon tissue (Figure 1e).

**Table 1:** Clinical characteristics of the three cases RA, rheumatoid arthritis; N.P., nothing particular; MTX, methotrexate; TA, tibialis anterior muscle; TAA, total ankle arthroplasty; ROM, range of motion; MMT, manual muscle testing; JSSF, Japanese Society for Surgery of the Foot

Variable	Case 1	Case 2	Case 3
Age, y	67	61	70
Sex	Female	Female	Female
Preoperative diagnosis	RA	RA	RA
Duration of disease (y)	25	33	2
Steinbrocker’s stage/functional class	Ⅳ/Ⅱ	Ⅳ/Ⅲ	Ⅳ/Ⅱ
Comorbidity	N.P.	Diabetes	N.P.
Prednisolone dose (mg/day)	None	7.0	None
MTX dose (mg/week)	None	None.	12.0
Biologics	Tocilizumab	Abatacept	None
Operation time (min)	170	175	243
Air tourniquet time (min)	129	137	227
Intraoperative complications	N.P.	N.P.	N.P.
When the TA tendon was exposed (days after TAA)	50	31	33
When the TA tendon was resected (days after TA exposed)	1	2	2
When the wound was healed (days after TA resected)	14	14	14
Active dorsiflexion ROM (Pre/Post-op 1 y, degrees)	0 / 0	5 / 5	0 / 5
Active plantarflexion ROM (Pre/Post-op 1 y, degrees)	5 / 15	15 / 20	30 / 25
JSSF RA foot ankle scale (Pre/Post-op 1 y)	38 / 69	53 / 74	56 / 68
JSSF ankle/hindfoot scale (Pre/Post-op 1 y)	41 / 67	35 / 84	38 / 74

Case 2

**Figure 2:**
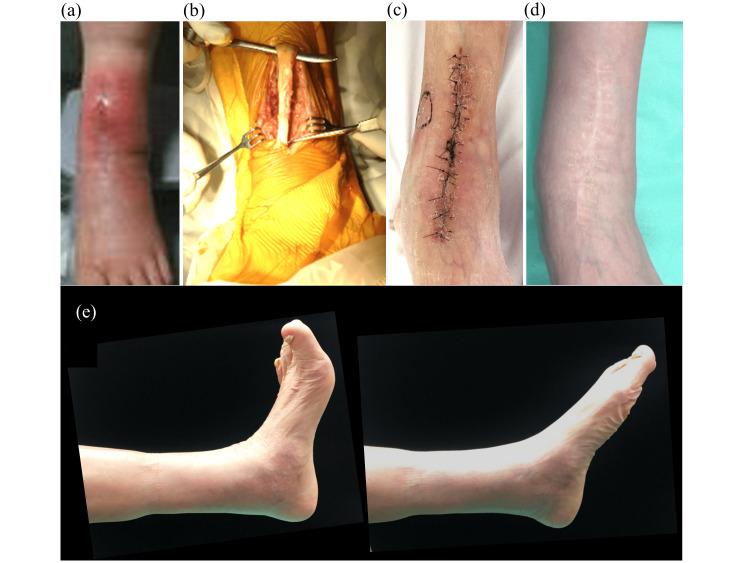
Case 2 (a) Cellulitis occurred 31 days after TAA, and the TA tendon was glimpsed with wound dehiscence. (b) The next day, a resection of the TA tendon was performed. The panel shows the preparation for the resection of the TA tendon. (c) Wound healing was complete, and the threads were removed 14 days after the resection. (d) The wound at three months after resection. No skin problems were observed. (e) Active dorsiflexion and plantarflexion of the ankle. TAA: total ankle arthroplasty, TA: tibialis anterior

In Case 2, A 61-year-old woman with a 33-year history of RA (Steinbrocker classification Class II stage IV) had a JSSF RA foot and ankle scale score of 53 points and a JSSF ankle/hindfoot scale score of 35 points (Table 1). Cellulitis was seen around the wound 31 days after TAA, and, at the same time, Staphylococcus aureus was detected in tendon cultures. Surgical findings and the wound healing process are shown in Figures 2a-2d. She had five degrees of active dorsiflexion, and the strength of the muscle for ankle dorsiflexion was maintained (MMT 5) (Figure 2e).

Case 3

In Case 3, A 70-year-old woman with a two-year history of RA (Steinbrocker classification Class II stage IV) had a JSSF RA foot and ankle scale score of 56 points and a JSSF ankle/hindfoot scale score of 38 points (Table 1). Cellulitis was seen around the wound 33 days after TAA, but no bacteria were detected in tendon cultures. Early resection of the TA tendon was performed, and then wound healing was achieved, as in the two other cases. In all three cases, wound healing was completed within two weeks after resection of the tendon.

## Discussion

It has been reported that complications associated with wound healing after TAA occur in 10-30% of cases [2,5]. Especially in RA patients, medications such as steroids and/or biologics can increase the risk of surgical site infection (SSI) [4] and delayed wound healing. Therefore, if the TA tendon is exposed to wound dehiscence after TAA, not only does it take a long time to achieve wound healing by conservative therapy, including negative pressure wound therapy (NPWT) and skin grafting but also there is a risk of progression to deep infection, especially in RA patients. A previous report showed that patients needed a skin flap procedure in 1.9% of primary TAAs because of wound problems, and then, among them, 21.1% of patients had flap failures [6]. While it is reported that a significantly decreased incidence of wound healing problems after primary total ankle arthroplasty was seen using incisional NPWT dressings [7,8]. So, NPWT would be also useful to be utilized for infectious wound dehiscence after TAA. However, during such repeated treatment, there is a possibility of deep infection. Thus, early resection of the tendon was considered proper for a tendon glimpse/exposure with wound dehiscence to prevent deep infection, especially in RA patients, because there was no critical dysfunction after tendon resection [2]. After TA tendon resection, other extensors, such as extensor hallux longus (EHL) and extensor digitorum longus (EDL), compensate for ankle dorsiflexion [2]. However, it is unclear how these compensations affect EHL/EDL in the long term. In short-term follow-up, previous reports demonstrated that surgical treatment for TA tendon rupture was sometimes associated with decreased dorsiflexion strength compared with the preoperative state, but there was no significant clinical difference between active young patients with reconstructive surgery and nonoperative elderly patients with low demand [9,10]. Taken together, in patients who have undergone TAA with weak muscle strength and relatively low activities of daily living, resection of the TA tendon is not considered problematic. Taken together, although there was a small number of cases in the present study, the wound healed quickly after early resection of the TA tendon, so early resection of the TA tendon is one of the effective treatments to shorten the period of wound healing, at the same time as avoiding deep infection. However, resection of the tendon is essentially undesirable, so modification of the skin incision/approach is needed to prevent tendon exposure with wound dehiscence. A modified anterolateral approach is now being tried and evaluated in our institutions. At the same time, Integra®, the collagen-glycosaminoglycan biodegradable matrix wound dressing, should also be tried because this matrix also provides coverage over exposed bones, tendons, cartilages, and joints.

In conclusion, although there were a small number of cases with short-term follow-up, early resection of the TA tendon appears to be a useful option for undesirable tendon exposure with infectious wound dehiscence to prevent deep infection and prolonged wound healing after total ankle arthroplasty in RA patients. However, further investigations with an increased number of cases and other novel procedures using NPWT and/or the novel biodegradable matrix wound dressing.

## Conclusions

In conclusion, although there were a small number of cases with short-term follow-up, early resection of the TA tendon appears to be a useful option for undesirable tendon exposure with wound dehiscence to prevent deep infection and prolonged wound healing after total ankle arthroplasty in RA patients.

## References

[1] Wood PL, Deakin S (2003). Total ankle replacement. The results in 200 ankles. J Bone Joint Surg Br.

[2] Etani Y, Ebina K, Hirao M (2020). A report of three cases which required tibialis anterior tendon resection to recover delayed wound healing after total ankle arthroplasty in patients with rheumatoid arthritis. Mod Rheumatol Case Rep.

[3] Ito H, Kojima M, Nishida K (2015). Postoperative complications in patients with rheumatoid arthritis using a biological agent - a systematic review and meta-analysis. Mod Rheumatol.

[4] Hislop H, Schwartz M (2007). Daniels and Worthingham's Muscle Testing: Techniques of Manual Examination. Saunders.

[5] Elliott AD, Roukis TS (2017). Anterior incision offloading for primary and revision total ankle replacement: a comparative analysis of two techniques. Open Orthop J.

[6] Gross CE, Garcia R, Adams SB, DeOrio JK, Easley ME, Nunley JA 2nd (2016). Soft tissue reconstruction after total ankle arthroplasty. Foot Ankle Int.

[7] Matsumoto T, Parekh SG (2015). Use of negative pressure wound therapy on closed surgical incision after total ankle arthroplasty. Foot Ankle Int.

[8] Sidorski A, Lundeen G (2020). The use of closed incision negative pressure therapy immediately after total ankle arthroplasty surgeries. Cureus.

[9] Markarian GG, Kelikian AS, Brage M, Trainor T, Dias L (1998). Anterior tibialis tendon ruptures: an outcome analysis of operative versus nonoperative treatment. Foot Ankle Int.

[10] Tickner A, Thorng S, Martin M, Marmolejo V (2019). Management of isolated anterior tibial tendon rupture: a systematic review and meta-analysis. J Foot Ankle Surg.

